# Supplementary material for: Abnormal proliferation of gut mycobiota contributes to the aggravation of Type 2 diabetes
Source: Commun Biol. 2023 Feb 28;6:226. doi: 10.1038/s42003-023-04591-x (PMC9974954; doi:10.1038/s42003-023-04591-x)
Supplement: Supplementary file 2 — Supplementary Information [file 42003_2023_4591_MOESM2_ESM.pdf]

Supplementary materials

Abnormal proliferation of gut mycobiota contributes to the aggravation of Type 2 diabetes

Li Bao[1,2], Ying Zhang[2,3], Guoying Zhang[4], Dechun Jiang[1,2], Dan Yan[2,3,✉]

[1] Department of Pharmacy, Beijing Shijitan Hospital, Capital Medical University, No.10 Tieyi-Road, Haidian District, Beijing, 100038, China.

[2] Beijing Key Laboratory of Bio-characteristic Profiling for Evaluation of Rational Drug Use, No.10 Tieyi-Road, Haidian District, Beijing, 100038, China.

[3] Beijing Friendship Hospital, Capital Medical University, No. 95 Yong'an Road, Xicheng District, Beijing, 100050, China.

[4] Biomedical Innovation Center, Beijing Shijitan Hospital, Capital Medical University, No.10 Tieyi-Road, Haidian District, Beijing, 100038, China.

✉Corresponding Author: Dan Yan, Tel: +86-10-63139318; E-mail: danyan@ccmu.edu.cn

Supplementary Table 1. The composition of the culture media used in this study.

	DIX (pH=6.2)	PDA (pH=5.6)	YPD (pH=6.2)
Agar	12g	15g	-
Pepton	6g	-	20g
Glucose	-	20g	20g
Potatoes extract	-	4g	-
Yeast extract	-	-	10g
Malt extract	36g	-	-
Ox-bile	20g	-	-
Tween 40	10ml	-	-
Glycerol	2ml	-	-
Oleic acid	2ml	-	-
Olive oil	2ml	-	-

DIX and PDA media were supplemented with 30mg/l of Imipenem, colistin and vancomycin, YPD broth was supplemented with 100ug/ml gentamicin and 100ug/ml chloramphenicol.

Supplementary Table 2. Related primers used in this study

	Name		Sequence (5’-3’)
1	Gapdh	Forward Primer	AGGTCGGTGTGAACGGATTTG
		Reverse Primer	TGTAGACCATGTAGTTGAGGTC
2	ZO-1	Forward Primer	ACCCGAAACTGATGCTGTGGAT
		Reverse Primer	AAATGGCCGGGCAGAACTTGTG
3	Occludin	Forward Primer	TTGAAAGTCCACCTCCTTACAG
		Reverse Primer	CCGGATAAAAAGAGTACGCTGG
4	irf5	Forward Primer	ATGCGGCTGGCAAGACTAC
		Reverse Primer	ACAGAGGCCGAATCTTATCCT
5	Syk	Forward Primer	CTACCTGCTACGCCAGAGC
		Reverse Primer	GCCATTAAGTTCCCTCTCGATG
6	Arg1	Forward Primer	CTCCAAGCCAAAGTCCTTAGAG
		Reverse Primer	GGAGCTGTCATTAGGGACATCA
7	ITS	ITS1	TCCGTAGGTGAACCTGCGG
		ITS4	TCCTCCGCTTATTGATATGC
8	Fungi-specific primers	1737F	GGAAGTAAAAGTCGTAACAAGG
		2043R	GCTGCGTTCTTCATCGATGC
9	*C. albicans*-specific Primers	CALB1	TTTATCAACTTGTCACACCAGA
		CALB2	ATCCCGCCTTACCACTACCG

Supplementary Table 3. Subject Characteristics.

	Gender	Age	BMI index	Fasting blood glucose (mmol/L)	HAbC1 (%)	Total cholesterol (mmol/L)	Total glyceride (mmol/L)
healthy volunteers n=15	F: 6 (40.00%)	36.86 ±10.04	22.84 ±3.07	4.58 ±0.44	--	4.80 ±0.61	1.07 ±0.31
	M: 9 (60.00%)						
T2DM n=26	F: 9 (34.62%)	47.92 ±10.32	25.43 ±3.66	10.40 ±3.08	9.64 ±2.13	4.86 ±0.69	2.31 ±1.49
	M: 17 (65.38%)						

* Plasma samples obtained from 15 healthy volunteers and 26 T2DM patients were used to detect 1,3-β-glucan levels, and stool samples from 6 healthy volunteers and 11 individuals with type 2 diabetes were performed to analysis the mycobiota with ITS2 sequence.

Supplementary Table 4. The fungal species isolated in this study.

	Fungal species	ITS sequencing results	CNKI Accession No.(>97%)
1	*Penicillium crustosum*	GAACCTGCGGAAGGATCATTACCGAGTGAGGGCCCTCTGGGTCCAACCTCCCACCC<br>GTGTTTATTTTACCTTGTTGCTTCGGCGGGCCCGCCTTAACTGGCCGCCGGGGGGCTT<br>ACGCCCCCGGGCCCGCGCCCGCCGAAGACACCCTCGAACTCTGTCTGAAGATTGAA<br>GTCTGAGTGAAAATATAAATTATTTAAAACTTTCAACAACGGATCTCTTGGTTCCGGC<br>ATCGATGAAGAACGCAGCGAAATGCGATACGTAATGTGAATTGCAAATTCAGTGAAT<br>CATCGAGTCTTTGAACGCACATTGCGCCCCCTGGTATTCCGGGGGGCATGCCTGTCC<br>GAGCGTCATTGCTGCCCTCAAGCCCGGCTTGTGTGTTGGGCCCCGTCCCCCGATCTC<br>CGGGGGACGGGCCCGAAAGGCAGCGGCGGCACCGCGTCCGGTCCTCGAGCGTATG<br>GGGCTTTGTCACCCGCTCTGTAGGCCCGGCCGGCGCTTGCCGATCAACCCAAATTTT<br>TATCCAGGTTGACCTCGGATCAGGTAGGGATACCCGCTG<br>AACTTAAGCATATCAA	MT316358.1
2	*Galactomyces pseudocandidus*	CTTCCGTAGGTGAACCTGCGGAAGGATCATTATGAATTATAAATATTTGTGAATTTACC<br>ACAGCAAACAAAAATCATACAATCAAAACAAAAATAATTAAAACTTTTAACAATGGA<br>TCTCTTGGTTCTCGTATCGATGAAGAACGCAGCGAAACGCGATATTTCTTGTGAATTG<br>CAGAAGTGAATCATCAGTTTTTGAACGCACATTGCACTTTGGGGTATCCCCCAAAGT<br>ATACTTGTTTGAGCGTTGTTTCTCTCTTGGAATTGCTTTGCTCTTCTAAAATTTCGAAT<br>CAAATTCGTTTGAAAAACAACACTATTCAACCTCAGATCAAGTAGGATTACCCGCTG<br>AACTTAAGCATATCAATAAGCGGAGGAAAAAG	NR_163519.1
3	*Geotrichum candidum*	GACCTGCGGAAGGATCATTATGAATTATAAATATTTGTGAATTTACCACAGCAAACAA<br>AAATCATACAATCAAAACAAAAATAATTAAAACTTTTAACAATGGATCTCTTGGTTCT<br>CGTATCGATGAAGAACGCAGCGAAACGCGATATTTCTTGTGAATTGCAGAAGTGAAT<br>CATCAGTTTTTGAACGCACATTGCACTTTGGGGTATCCCCCAAAGTATACTTGTTTGA<br>GCGTTGTTTCTCTCTTGGAATTGCTTTGCTCTTCTAAAATTTCGAATCAAATTCGTTTG<br>AAAAACAACACTATTCAACCTCAGATCAAGTAGGATTACCCGCTGAACTTAAGCATA<br>TCAAAAAGCGGGAGGAATGG	MK886527.1
4	*Candida glabrata*	GGCCTTCGGATTTATTGATTTGTCTGAGCTCGGAGAGAGACATCTCTGGGGAGGACC<br>AGTGTAGACACTCAGGAGGTTCCTAAAATATTTTCTCTGCTGTGAATGCCATTTCTCC<br>TGCCTGCGCTTAAGTGCGCGGTTGGTGGGTGTTCTGCAGTGGGGGGAGGGAGCCGA<br>CAAAGACCTGGGAGTGTGCGTGGATCTCTCTATTCCAAAGGAGGTGTTTTATCACAC	NR_130691.1

		GACTCGACACTTTCTAATTACTACACACAGTGGAGTTTACTTTACTACTATTCTTTTGTTCGTTGGGGGAACGCTCTCTTTCGGGGGGGGAGTTCTCCCAGTGGATGCAAACACAAACAAATATTTTTTTAAACTAATTCAGTCAACACAAGATTTCTTTTAGTAGAAAACAACTTCAAAACTTTCAACAATGGATCTCTTGGTTCTCGCATCGATGAAGAACGCAGCGAAATGCGATACGTAATGTGAATTGCAGAATTCCGTGAATCATCGAATCTTTGAACGCACATTGCGCCCTCTGGTATTCCGGGGGGCATGCCTGTTTGAGCGTCATTTCCTTCTCAAACACATTGTGTTTGGTAGTGAGTGATACTCTCGTTTTTGAGTTAACTTGAAATTGTAGGCCATATCAGTATGTGGGACACGAGCGCAAGCTTCTCTATTAATCTGCTGCTCGTTTGCGCGAGCGGCGGGGGTTAATACTGTATTAGGTTTTACCAACTCGGTGTTGATCTAGGGAGGGATAAGTGAGTGTTCTGTGCGTGCTGGGCAGACAGACGTCTTTAAGTTGACCTCAAATCAGGTAGGTGCCGGATTT	
5	*Arthrinium kogelbergense*	AACCTGCGGAGGGATCATTACAGAGTTATACAACTCCCATACCATCTGTTAACCTACCCAGTTATGCCTCGGCGTAAGCTCGGTTGGAGGCACCTGCAGCTACCCTGTAGTTGCGGACTGCCAACTCCAGCCGCGGCCCGCCGGCGGTACACTAAACTCTGTTTTATTTTATATTCTGAGCGTCTTATTTTAATAAGTTAAAACTTTCAACAACGGATCTCTTGGTTCTGGCATCGATGAAGAACGCAGCGAAATGCGATAAGTAATGTGAATTGCAGAATTCAGTGAATCATCGAATCTTTGAACGCACATTGCGCCCATCAGTATTCTGGTGGGCATGCCTGTTCGAGCGTCATTTCAACCCTTAAGCCTAGCTTAGTGTTGGGAATCTGCTGTACTGCAGTTCCTTAAAGACAGTGGCGGAGCGGCGGTAGTCCTCTGAGCGTAGTAATTTATTTCTCGCTTTTGTCAGGCTCTGTCCTCCCGCCATAAAACCCCCAATTTTTTAGTGGTTGACCTCGGATCAGGTAGGAATACCCGCTGAACTTAAGCATATCAATAAGCCGGAG	NR_120272.1
6	*Aspergillus versicolor*	CCTGCGGAAGGATCATTACTGAGTGCGGGCTGCCTCCGGGCGCCCAACCTCCCACCCGTGAATACCTAACACTGTTGCTTCGGCGGGGAACCCCCTCGGGGGCGAGCCGCCGGGGACTACTGAACTTCATGCCTGAGAGTGATGCAGTCTGAGTCTGAATATAAAATCAGTCAAAACTTTCAACAATGGATCTCTTGGTTCCGGCATCGATGAAGAACGCAGCGAACTGCGATAAGTAATGTGAATTGCAGAATTCAGTGAATCATCGAGTCTTTGAACGCACATTGCGCCCCCTGGCATTCCGGGGGGCATGCCTGTCCGAGCGTCATTGCTGCCCATCAAGCCCGGCTTGTGTGTTGGGTCGTCGTCCCCCCCGGGGGACGGGCCCGAAAGGCAGCGGCGGCACCGTGTCCGGTCCTCGAGCGTATGGGGCTTTGTCACCCGCTCGACTAGGGCCGGCCGGGCGCCAGCCGACGTCTCCAACCATTTTTCTTCAGGTTGACCTCGGATCAGGTAGGGATACCCGCTGAACTTAAGCATATCA	NR_131277.1
7	*Candida*	CATTGCCTTATTGCACCACATGTGTTTTTCTTTGAACAAACTTGCTTTGGCGGTGGGC	NR_138276.1

	albicans	CCAGCCTGCCGCCAGAGGTCTAAACTTACAACCAATTTTTTATCAACTTGTCACACC<br>AGATTATTACTAATAGTCAAAACTTTCAACAACGGATCTCTTGGTTCTCCCATCCATG<br>AAGAACGCAGCGAAATGCGATACGTAATATGAATTGCAGATATTCGTGAATCATCCAA<br>TCTTTGAACGCACATTGCGCCCTCTGGTATTCCGGAGGGCATGCCTGGTTGAGCGTC<br>GTTTCTCCCTCCAACCGCTGGGTTTGGTGGTGAGCAATACGACTTGGGTTGCTTGAA<br>AGACGGAGTGGAAGGGGGGATCGCTTTGAAATGGCTTAGTCTAACCAAAAACATTG<br>CTTGCGGCGGAACGTCTACCACGTATATCTTCAACTTTGACCTCAATCAGGGAGGAC<br>TACCCGCTGAACTTAAGCTATCAATAAGCGGAGGAAAAATTTGTTGATAT	

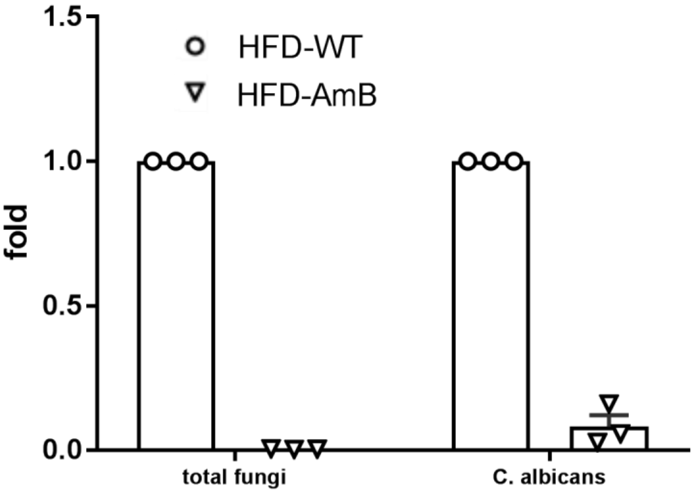

Supplementary Figure 1. Fecal total fungi and total *C. albicans* in Amphotericin B treated mice were assessed by qPCR (n=3).

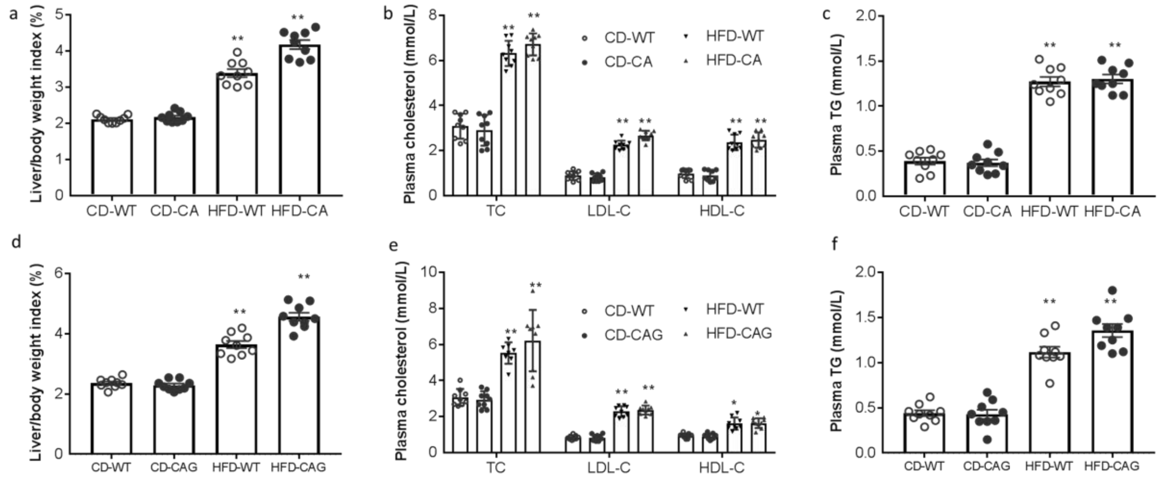

Supplementary Figure 2. Effects of *C. albicans* strains and CAG on the blood dyslipidemia. (a) and (d) liver/body weight index, (b) and (e) Plasma total cholesterol, LDL-C and HDL-C, (c) and (f) Plasma triglyceride. Data are presented as the mean ± standard error of the mean (SEM); N = 9 mice per group. Statistical analysis was done using oneway ANOVA followed by the Tukey post hoc test: (∗) $P < 0.01$, (∗∗) $P < 0.01$ vs CD-WT group.

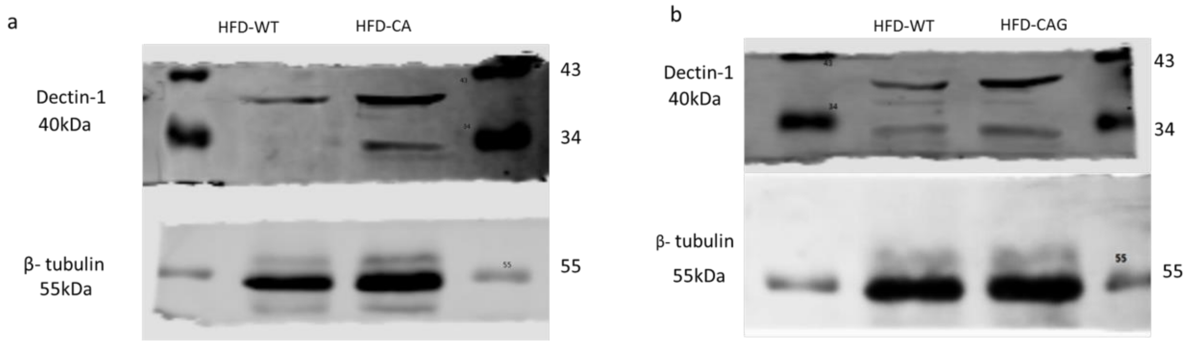

Supplementary Figure 3. The colon dectin-1 level of the C. albicans-treated or CAG-treated mice (uncropped images, refered to Extended Fig 6a-b).
