# Supplementary material for: Abnormal proliferation of gut mycobiota contributes to the aggravation of Type 2 diabetes
Source: Commun Biol. 2023 Feb 28;6:226. doi: 10.1038/s42003-023-04591-x (PMC9974954; doi:10.1038/s42003-023-04591-x)
Supplement: Supplementary file 3 — Description of Additional Supplementary Data [file 42003_2023_4591_MOESM3_ESM.docx]

**Description of Additional Supplementary Files**

**File name:** Supplementary Data 1

**Description:** The source data behind the figures in the paper.
