# Supplementary material for: Abnormal proliferation of gut mycobiota contributes to the aggravation of Type 2 diabetes
Source: Commun Biol. 2023 Feb 28;6:226. doi: 10.1038/s42003-023-04591-x (PMC9974954; doi:10.1038/s42003-023-04591-x)
Supplement: Supplementary file 5 — nr-reporting-summary [file 42003_2023_4591_MOESM5_ESM.pdf]

nature portfolio

Corresponding author(s): COMMSBIO-22-0564C

Last updated by author(s): Feb 2, 2023

Reporting Summary

Nature Portfolio wishes to improve the reproducibility of the work that we publish. This form provides structure for consistency and transparency in reporting. For further information on Nature Portfolio policies, see our Editorial Policies and the Editorial Policy Checklist.

Statistics

For all statistical analyses, confirm that the following items are present in the figure legend, table legend, main text, or Methods section.

n/a	Confirmed	
☐	☒	The exact sample size (*n*) for each experimental group/condition, given as a discrete number and unit of measurement
☐	☒	A statement on whether measurements were taken from distinct samples or whether the same sample was measured repeatedly
☐	☒	The statistical test(s) used AND whether they are one- or two-sided<br>*Only common tests should be described solely by name; describe more complex techniques in the Methods section.*
☐	☒	A description of all covariates tested
☒	☐	A description of any assumptions or corrections, such as tests of normality and adjustment for multiple comparisons
☒	☐	A full description of the statistical parameters including central tendency (e.g. means) or other basic estimates (e.g. regression coefficient) AND variation (e.g. standard deviation) or associated estimates of uncertainty (e.g. confidence intervals)
☒	☐	For null hypothesis testing, the test statistic (e.g. *F*, *t*, *r*) with confidence intervals, effect sizes, degrees of freedom and *P* value noted<br>*Give P values as exact values whenever suitable.*
☒	☐	For Bayesian analysis, information on the choice of priors and Markov chain Monte Carlo settings
☒	☐	For hierarchical and complex designs, identification of the appropriate level for tests and full reporting of outcomes
☒	☐	Estimates of effect sizes (e.g. Cohen's *d*, Pearson's *r*), indicating how they were calculated

Our web collection on statistics for biologists contains articles on many of the points above.

Software and code

Policy information about availability of computer code

Data collection	no software was used to collect data
Data analysis	GraphPad 6.0

For manuscripts utilizing custom algorithms or software that are central to the research but not yet described in published literature, software must be made available to editors and reviewers. We strongly encourage code deposition in a community repository (e.g. GitHub). See the Nature Portfolio guidelines for submitting code & software for further information.

Data

Policy information about availability of data

All manuscripts must include a data availability statement. This statement should provide the following information, where applicable:

- Accession codes, unique identifiers, or web links for publicly available datasets
- A description of any restrictions on data availability
- For clinical datasets or third party data, please ensure that the statement adheres to our policy

All data generated or analyzed during this study are included in this published article (and its supplementary information files).

Human research participants

Policy information about studies involving human research participants and Sex and Gender in Research.

Reporting on sex and gender	As stated in Table 1 of the supplementary material, 60% of the healthy volunteers in our study were male and 40% were female, and 65.38% of the type 2 diabetes patients were male and 34.62% were female.
Population characteristics	As stated in Table 1 of the supplementary document, the average age of healthy volunteers in our study is 36.86 years old, with a BMI of 22.84 and fasting blood glucose of 4.58; The average age of patients with type 2 diabetes was 47.92 years old, BMI index was 25.43, and fasting blood glucose was 10.40.
Recruitment	All volunteers are publicly recruited, and there is no selection bias
Ethics oversight	The study was approved by the Ethics Committee of Beijing Shijitan Hospital Affiliated to Capital Medical University (2017-035) and registered on the Chinese Clinical Trial Registry (ChiCTR2100042049), as well as conducted under the guidelines of the Helsinki Declaration

Note that full information on the approval of the study protocol must also be provided in the manuscript.

Field-specific reporting

Please select the one below that is the best fit for your research. If you are not sure, read the appropriate sections before making your selection.

☒ Life sciences ☐ Behavioural & social sciences ☐ Ecological, evolutionary & environmental sciences

For a reference copy of the document with all sections, see nature.com/documents/nr-reporting-summary-flat.pdf

Life sciences study design

All studies must disclose on these points even when the disclosure is negative.

Sample size	In the animal experiment, the mice were randomly divided into 8-9 mice in each group. Under the premise of statistical validity, it conforms to the 4R principle.
Data exclusions	no data was excluded
Replication	All attempts are replication were successful
Randomization	it was randomly grouped.
Blinding	It is not a double-blind collection, because we did the comparison between the two groups of intestinal fungi of healthy volunteers and patients with type 2 diabetes, and we need to strictly control the selection conditions.

Reporting for specific materials, systems and methods

We require information from authors about some types of materials, experimental systems and methods used in many studies. Here, indicate whether each material, system or method listed is relevant to your study. If you are not sure if a list item applies to your research, read the appropriate section before selecting a response.

Materials & experimental systems

n/a	Involved in the study	
☐	☒	Antibodies
☒	☐	Eukaryotic cell lines
☒	☐	Palaeontology and archaeology
☐	☒	Animals and other organisms
☐	☒	Clinical data
☒	☐	Dual use research of concern

Methods

n/a	Involved in the study	
☒	☐	ChIP-seq
☒	☐	Flow cytometry
☒	☐	MRI-based neuroimaging

Antibodies

Antibodies used	Alexa Fluor 790 goat anti-rabbit IgG H&L (ab186697; Abcam, Cambridge, UK) , Alexa Fluor 680 goat anti-mouse IgG (ab186694; Abcam, Cambridge, UK), DECTIN-1 (GB111816, Servicebio), beta-tubulin (AF7018, Affinity)
Validation	DECTIN-1 was expressed in liver, spleen and thymus of human, mouse and rat. It was also known as CLEC7A, is a member of the C-type lectin/C-type lectin-like domain superfamily.

Animals and other research organisms

Policy information about studies involving animals; ARRIVE guidelines recommended for reporting animal research, and Sex and Gender in Research

Laboratory animals	6-week-old C57BL/6J male mice were used in this study.
Wild animals	this study did not involve the wild animals.
Reporting on sex	In the analysis of human intestinal fungi, the sex ratio is nearly the same, and there is no significant difference in result. In animal experiments, we use male mice, because we all know that the influence of female hormones complicates many factors.
Field-collected samples	This study did not involve samples collected from the field
Ethics oversight	The study was approved by the Ethics Committee of Beijing Shijitan Hospital Affiliated to Capital Medical University (2017-035)

Note that full information on the approval of the study protocol must also be provided in the manuscript.

Clinical data

Policy information about clinical studies

All manuscripts should comply with the ICMJE guidelines for publication of clinical research and a completed CONSORT checklist must be included with all submissions.

Clinical trial registration	this study was registered on the Chinese Clinical Trial Registry (ChiCTR2100042049)
Study protocol	The full trial protocol are included in this published article (and its supplementary information files).
Data collection	from 2021-1-20 to 2021-3-31
Outcomes	This study is mainly to discover the difference of intestinal fungi between healthy volunteers and type 2 diabetic patients, and then to verify the function of intestinal fungi with significant difference. The main measured index were blood glucose and glucose tolerance.
